# Miniemulsion copolymerization of (meth)acrylates in the presence of functionalized multiwalled carbon nanotubes for reinforced coating applications

**DOI:** 10.3762/bjnano.8.134

**Published:** 2017-06-27

**Authors:** Bertha T Pérez-Martínez, Lorena Farías-Cepeda, Víctor M Ovando-Medina, José M Asua, Lucero Rosales-Marines, Radmila Tomovska

**Affiliations:** 1 POLYMAT and Departamento de Química Aplicada, Facultad de Ciencias Químicas, University of the Basque Country UPV/EHU, Joxe Mari Korta zentroa, Tolosa Etorbidea 72, Donostia-San Sebastián 20018, Spain; 2 Departamento de Ingeniería Química, Universidad Autónoma de Coahuila, Blvd. V. Carranza e Ing. José Cárdenas V. S/N, Saltillo, Coah, 25280 México; 3 Ingeniería Química, Coordinación Académica Región Altiplano (COARA) Universidad Autónoma de San Luis Potosí, Carretera a Cedral KM 5+600, San José de las Trojes, Matehuala, SLP, 78700 México,; 4 IKERBASQUE, Basque Foundation for Science, Bilbao, Spain

**Keywords:** electrical conductivity, hybrid polymers, mechanical properties, miniemulsion polymerization, multiwalled carbon nanotubes

## Abstract

Film forming, stable hybrid latexes made of methyl metacrylate (MMA), butyl acrylate (BA) and 2-hydroxyethyl methacrylate (HEMA) copolymer reinforced with modified multiwalled carbon nanotubes (MWCNTs) were synthesized by in situ miniemulsion polymerization. The MWCNTs were pretreated by an air sonication process and stabilized by polyvinylpyrrolidone. The presence of the MWCNTs had no significant effect on the polymerization kinetics, but strongly affected the polymer characteristics (*T*_g_ and insoluble polymer fraction). The performance of the in situ composites was compared with that of the neat polymer dispersion as well as with those of the polymer/MWCNT physical blends. The in situ composites showed the presence of an additional phase likely due to the strong interaction between the polymer and MWNCTs (including grafting) that reduced the mobility of the polymer chains. As a result, a substantial increase of both the storage and the loss moduli was achieved. At 60 °C, which is above the main transition region of the polymer, the in situ composites maintained the reinforcement, whereas the blends behaved as a liquid-like material. This suggests the formation of a 3D network, in good agreement with the high content of insoluble polymer in the in situ composites.

## Introduction

Carbon nanotubes (CNTs) are hollow, fiber-like materials, with a diameter on the nanometer scale and a relatively long length on the micrometer scale, resulting in a very high aspect ratio material. Two types of CNTs exist, those made of a single graphene layer rolled-up into a cylinder (single-walled carbon nanotubes (SWCNTs)) or multiwalled CNTs (MWCNTs) that consist of two or more sheets of graphene concentrically rolled around a hollow core. Due to the excellent electrical, optical, thermal, mechanical, and chemical properties of CNTs, they are considered to be an advanced material that may be useful for multiple applications, one of which is polymer composite synthesis [1–4].

By inclusion of CNTs in polymer matrices, nanostructured materials with improved mechanical, electrical and thermal properties may be synthesized. The interaction between the polymer and the CNTs is crucial to principally determine the distribution of CNTs within the polymer matrix and to obtain the best performance from the nanocomposites [1–2]. One way to improve this interaction is to functionalize the surface of CNTs, either by covalent attachment or through the supramolecular adsorption or wrapping of suitable functionalities and even surface active substances [1–2,4–5].

Various techniques have been developed for the synthesis of CNT–polymer composites, including solution mixing [6–7], melt blending [8–12], latex technology (blends of latexes and CNT dispersions) [13–17], and in situ polymerization [8,12,18–21]. In situ polymerization can be performed in solution, bulk and in dispersed media. Polymerization in dispersed media allows a relatively easy control of the reactor temperature (which is a drawback of bulk polymerization), and when the continuous medium is water, the process is much more environmentally friendly than solution polymerization. Furthermore, this technique has the potential of offering a better distribution of CNTs in the film cast from the dispersion because the CNTs are placed in the interstitial sites between the polymer nanoparticles, which hinders CNT aggregation in the film.

Emulsion polymerization is the most frequently used waterborne polymerization process in industry [22–26]. However, especially for hybrid systems that contain an additional solid phase, miniemulsion polymerization is much more versatile [27–31]. The characteristic feature of this process is that particle formation predominantly occurs by nucleation of the preformed miniemulsion droplets, which minimizes the changes in the system during the particle nucleation period and does not require massive diffusion of the components of the formulation through the aqueous phase. Ham et al. [32–33] used a so-called miniemulsion process in an attempt to cover SWCNTs with polystyrene nanoparticles, where *n*-pentanol was used as a hydrophobe to minimize Ostwald ripening. However, *n*-pentanol is rather water soluble and it cannot hinder Ostwald ripening. Therefore, it is doubtful that the monomer droplets were stable. Ha et al. [18] polymerized miniemulsions prepared by sonicating a mixture of surfactant-stabilized SWCNTs, monomers (styrene and isoprene) and a costabilizer (hexadecane), finding that the surfactant was transferred to the latex during the reaction; this led to nanotube aggregation. Donescu et al. [34] carried out the miniemulsion polymerization of styrene, styrene/acrylonitrile and methyl methacrylate (MMA) in the presence of MWCNTs. Grafting of the polymer on the MWCNTs was reported. The resulting nanocomposites were foamed with supercritical CO_2_. The foams showed a decreased pore size, an increased cell density and higher volume expansion when the MWCNT concentration increased. Capek and Kocsisova [35] studied the effect of the type and concentration of surfactant on the kinetics of miniemulsion polymerization of butyl acrylate (BA) in the presence of CNTs.

Waterborne polymer dispersions are mainly used for coatings and adhesives, which involve the formation of films directly cast from the dispersion, usually at ambient temperature [22,24]. This limits the potential application of the dispersions prepared in the works discussed above [18,32–34] because high glass transition temperature (*T*_g_) polymers that do not form films at ambient temperature were synthesized. From BA dispersions, the adhesive films might eventually be prepared; however, Capek and Kocsisová [35] did not study this.

The main aim of this work is to produce film-forming waterborne composites for reinforced coating applications, in which the reinforcement is achieved by addition of small amounts of MWCNTs. The synthesis was carried out by miniemulsion co-polymerization of MMA/BA/2-hydroxyethyl methacrylate (HEMA) in the presence of MWCNTs. The minor amount of HEMA in the monomer mixture was added to further improve the interaction between the polymer and the MWCNTs. The disentanglement of the MWCNTs bundles prior to use in polymer composites was performed by ultrapower sonication performed either in water or in air, and afterwards stabilization by polyvinylpyrrolidone (PVP) in dispersion. Air-sonicated MWCNTs allowed for a smooth polymerization reaction, resulting in 20 wt % solids content (s.c.), stable and film-forming latexes with up to 1 wt % MWCNTs incorporated. Important mechanical and thermal reinforcement was achieved due to the 3D network formation of the filler within the polymer matrix and creation of strong interactions (including grafting between the phases).

## Experimental

### Materials

Multiwalled carbon nanotubes (MWCNTs, length = 5–10 µm; diameter = 10–20 nm) were purchased from IoLiTec Nanomaterials Co. (98.5%, Germany). Polyvinylpyrrolidone (PVP) with molar mass of 10,000 g·mol^−1^ was purchased from Sigma-Aldrich (99%). Potassium persulfate (KPS) was used as an initiator and purchased from Fluka. Sodium dodecyl sulfate (SDS) from Sigma-Aldrich (99%) and stearyl acrylate (SA) from BASF (98%), were used as surfactant and costabilizer, respectively. MMA monomer was acquired from Sigma-Aldrich (>98.5%), BA and HEMA were purchased from Fluka (>99%) and used as received. Double deionized water was used throughout the experiment.

### MWCNT pretreatment

MWCNTs were pretreated by sonication in air, after which they were dispersed in water in presence of PVP. The procedure for sonication in air was as follow: 0.35 g of MWCNTs were placed into a 50 mL beaker that had a magnetic stirrer. An ultrasound tip (Branson 450 instrument, Danbury, CT) was introduced into the beaker (keeping a separation between the ultrasound tip and magnetic stirrer of approximately 2 cm and 1 cm separation between the tip and the MWCNTs) and the beaker was sealed. Afterwards, ultrasound was applied for 1.5 h at 70% of power output and 50% duty cycle under magnetic stirring (200 rpm). In addition to disentangling the bundles, sonication is expected to break the MWCNTs.

The aqueous dispersion of MWCNTs used in the composite preparation was prepared by dispersing the treated MWCNTs (0.15 g) in water (50.5 g) in the presence of polyvinylpyrrolidone (PVP, 3 g) and sonicated for 10 min (70% of power output and 50% duty cycle).

### Miniemulsion polymerization of MMA/BA/HEMA in the presence of MWCNTs

Batch miniemulsion polymerization samples were made in a 150 mL glass-jacketed reactor equipped with mechanical stirring (200 rpm). The organic phase contained 20 g of a mixture of MMA/BA/HEMA/SA (47.6/47.6/0.96/3.84 wt/wt or 54.76/43.21/0.6/1.44 mol/mol). SA was used as a costabilizer to prevent the Oswald ripening process.

It is worth pointing out that SA was incorporated into the copolymer; therefore, strictly speaking, a MMA/BA/HEMA/SA copolymer was formed. The aqueous phase was formed by mixing 40 g of water with 0.2 g SDS. The aqueous and the organic phases were mixed under vigorous stirring and this mixture was sonicated under magnetic stirring for 15 min at 9 output control and 80% duty cycle with a Branson 450 instrument (Danbury, CT). Sonication was carried out in an ice bath to avoid overheating. After miniemulsion preparation, it was mixed under stirring (250 rpm, 15 min) with different amounts the aqueous dispersion of PVP-stabilized MWCNTs (0.1, 0.25, 0.5, 0.75 and 1.0 wt % with respect to monomers).

The resulting miniemulsion was bubbled with N_2_ for 15 min and the temperature was raised to 70 °C. Afterwards, 0.20 g of KPS (1 wt % with respect to monomers) were added to the reactor to start the polymerization. The N_2_ flow was maintained throughout polymerization.

Blank polymer latexes were synthesized by miniemulsion polymerization using the same organic phase and 40 g of water with 0.2 g of SDS. The mixture was under agitation for 15 min and then sonicated under magnetic stirring for 15 min (80% of duty cycle, 9 output control, Branson 450). Sonication was carried out in an ice bath to avoid overheating. Polymerization was carried out at 70 °C using KPS.

### Films

Films from the hybrid latexes MMA/BA/HEMA/MWCNT and from the blends were cast on Teflon molds and dried in a constant climate chamber (Espec Bench SH-641) at 25 °C and 80% of relative humidity for 3 days. Table 1 presents the nomenclature and characteristics of all the samples investigated throughout this study.

**Table 1:** Preparation method and characteristics of the investigated samples.

Sample	Preparation method	Weight fraction of MWCNTs (wt %)^a^

blank polymer	miniemulsion polymerization of neat monomers	0
in situ 0.5 wt %	miniemulsion polymerization in presence of MWCNTs	0.5
in situ 1.0 wt %	miniemulsion polymerization in presence of MWCNTs	1.0
blend 0.5 wt %	mixing of blank polymer + MWCNTs aqueous dispersion	0.5
blend 1.0 wt %	mixing of blank polymer + MWCNTs aqueous dispersion	1.0
aged in situ 0.5%	in situ 0.5 wt % film stored for three years	0.5
aged blend 0.5%	blend 0.5 wt % film stored for three years	0.5

^a^Weight percent based on monomer.

### Characterization

The conversion process was performed gravimetrically [36]. Latex stability was studied by measuring the light backscattered from the dispersions using a Turbiscan Lab expert apparatus scanning the dispersions placed in a vial (55 mm path length) at regular intervals. The particle size was measured by quasielastic light scattering (QLS) with a ZEN1600 apparatus (Malvern Instruments). The samples were prepared by diluting one drop of latex in deionized water. The reported diameters are the average of two subsequent measurements. It should be noted that the content of MWCNTs after dilution is below the detection limit of the apparatus so their presence did not affect the measurements. An insoluble fraction in tetrahydrofuran (THF) of the composite (gel content) was determined by the Soxhlet extraction.

The fractured composite films were prepared under liquid nitrogen and scanning electron microscopy (SEM) images were taken in a Hitachi S-4800. Differential scanning calorimetry (DSC) measurements of films cast from hybrid latexes and blends were carried out in a Q1000, TA Instruments apparatus. 5 mg of each sample were placed in standard aluminum DSC pans and analyzed in air atmosphere at a heating rate of 10 °C/min, starting from cooling to 80 °C and heating to 120 °C. The results of the second heating scan are reported. The conductivity of films was measured using a four-point probe (Digital Lock-In, SR850), and the viscoelastic properties of the films were determined in a dynamic mechanical thermal analyzer (DMTA, Triton Technology, Tritec 2000 DMTA). The scans were performed at a frequency of 1 Hz with a heating rate of 4 °C min^−1^ and the storage and loss moduli were measured. The measurements were run in single-cantilever bending mode with a displacement of 0.005 mm and a length between the clamps of 2 mm.

The mechanical properties of the films were determined by tensile test measurement. The films with an average thickness of 450 µm were prepared by drying in Teflon molds at 25 °C and 80% humidity for 3 days. The measurements were performed in an MTS Insight 10 instrument at a constant strain velocity of 2 mm·s^−1^ at two different temperatures, 25 °C and 60 °C.

## Results and Discussion

### Miniemulsion polymerization kinetics and properties of the hybrid latexes

In situ miniemulsion polymerization at 20 wt % solid content was performed in the presence of various amounts of air-sonicated MWCNTs (0–1 wt % with respect to monomer) stabilized by PVP. All the latexes had a dark blue color and were very stable (Figure S1, Supporting Information File 1), presenting less than 1 wt % coagulum after the reaction.

Table 2 presents the characteristics of the miniemulsions and the corresponding in situ latexes prepared with air-sonicated MWCNTs. It can be seen that the droplet size increased with increased MWCNT concentration. A possible reason for this finding is that, as the total amount of surfactant (SLS and PVP) used in these experiments was constant, the amount of surfactant available for droplet stabilization decreased as the MWCNT concentration increased. The concentration of MWCNTs may also have an influence on the miniemulsification process, which is sensitive to changes in viscosity [37–38].

**Table 2:** Droplet *z*-average diameter (*d*_d_) and particle *z*-average diameter (*d*_p_), number of particles (*N*_p_), and gel content in the final latexes obtained in the miniemulsion polymerization of MMA/BA/HEMA with different MWCNT concentrations.

MWCNT (wt %)	*d*_d_ (nm)	*d*_p_ (nm)	*N*_p_ (number/*L*)	Weight fraction of insoluble polymer (%)

0	40	70	1.11 × 10^18^	0
0.10	100	101	3.71 × 10^17^	13
0.25	210	102	3.60 × 10^17^	21
0.50	246	97	4.20 × 10^17^	30
0.75	261	98	4.06 × 10^17^	45
1.0	283	99	3.94 × 10^17^	85

**Figure 1:**
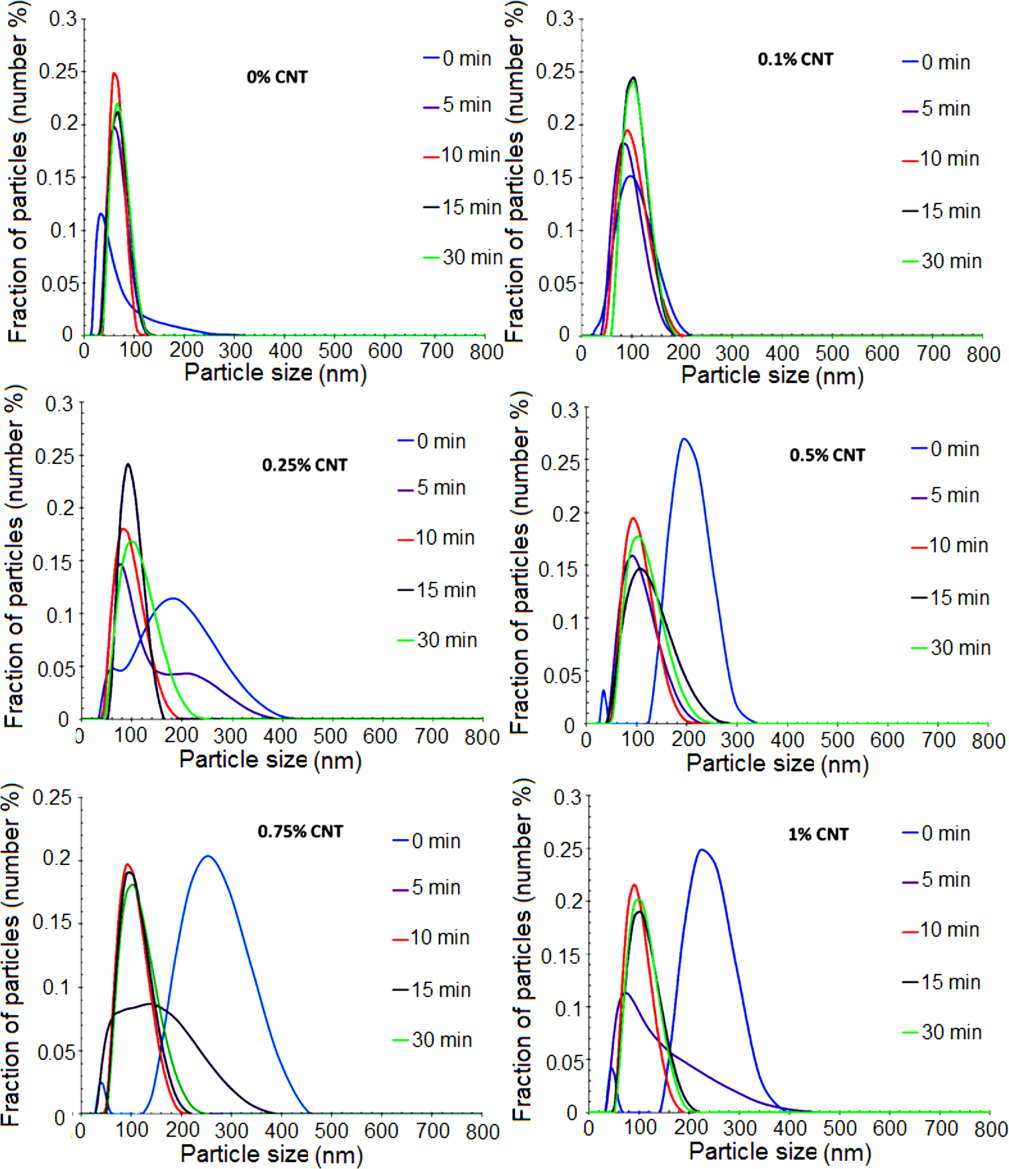
Particle size distribution in miniemulsion polymerization of MMA/BA/HEMA in the presence of various amounts of functionalized MWCNTs at different reaction times.

The comparison between the droplet and particle diameters (Table 2) may shed some light on the relative importance of these effects. It can be seen that a significant secondary nucleation occurred and that the final particle size was not affected by the concentration of MWCNTs. This suggests that the number of polymer particles was controlled by the surfactant available, which was independent of the MWCNT concentration, indicating that the presence of MWCNTs reduced the efficiency of the miniemulsification by increasing the viscosity of the system. In Figure 1, the evolution of the particle size distribution per number of particles during the miniemulsion polymerization of MMA/BA/HEMA in the presence of various quantities of MWCNTs (0.1–1%) is presented.

It can be seen that, except for the 0.1 wt % MWCNT sample, most of the particles were formed by secondary nucleation likely due to the combined effect of the presence of a highly water soluble monomer (HEMA) that promoted the formation of oligomers in the aqueous phase, and the large droplet size that reduced the total surface area of the droplets and consequently their ability to capture oligomers from the aqueous phase. For the 0.1 wt % sample, the particle size was similar to the droplet size, likely because the smaller droplets had a larger surface area, and hence they were more efficient capturing radicals from the aqueous phase.

**Figure 2:**
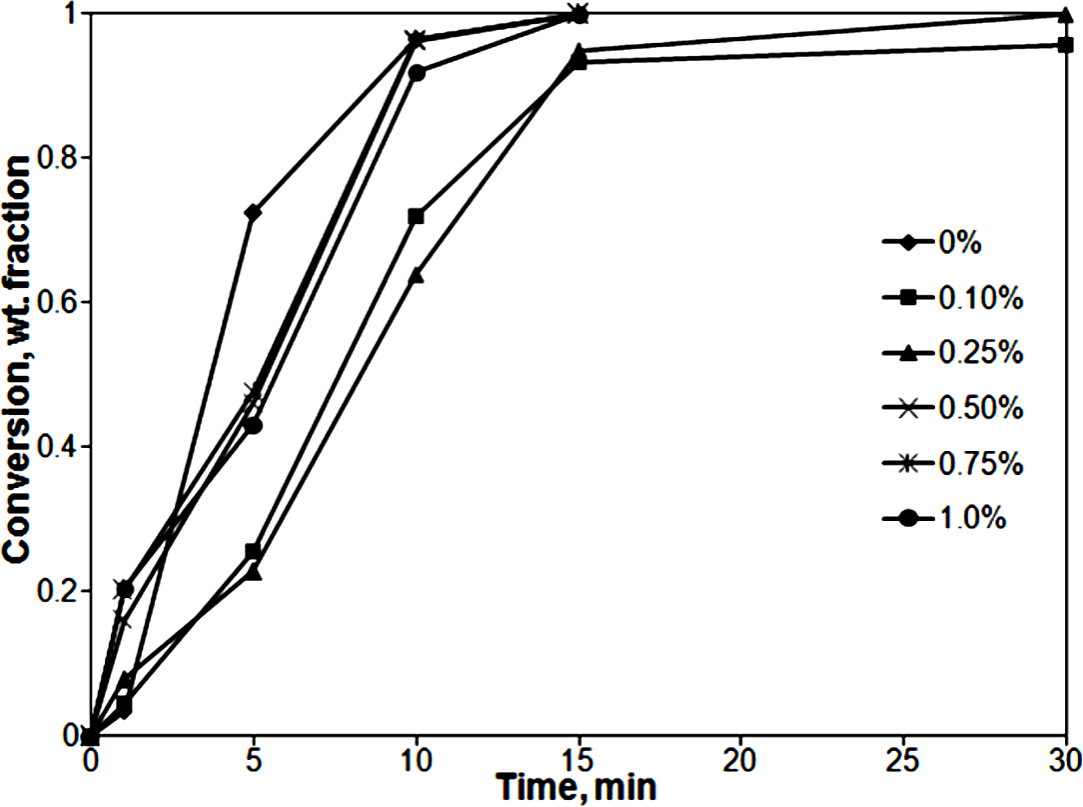
Conversion vs time curves for the MMA/BA/HEMA miniemulsion polymerizations with different MWCNT concentrations. Continuous lines are a guide to the eye.

Figure 2 presents the kinetics of the miniemulsion polymerizations carried out with different air-sonicated MWCNT loads. In all cases, final conversion yields between 96% and 100% were obtained after 30 min of polymerization. It can be seen that, after the initial stages, all the reactions carried out in the presence of MWCNTs presented almost the same polymerization rate (slope of the curve conversion vs time), which agrees with the similar number of particles. The polymerization carried out in the absence of MWCNTs showed a higher polymerization rate that agrees well with a higher number of polymer particles. The discrepancies at shorter process times are common in batch processes carried out using technical monomers (monomers containing inhibitors).

Table 2 also shows that the fraction of insoluble polymer in THF (often called gel) increased with the concentration of MWCNTs, reaching values as high as 85% for 1 wt % CNTs. In this regard, it is worth pointing out that this fraction was measured in films, not in individual particles. On the other hand, the blank experiment (first row in Table 2) shows that, in agreement with previous results [39], no gel was obtained for the monomer composition used.

The substantial increase in the fraction of insoluble polymer with slight increase of MWCNT load could be explained by two different processes. On one hand, polymer chains may be grafted onto either the PVP or the surface of MWCNTs, as it has been reported in the emulsion polymerization of styrene initiated with KPS in the presence of MWCNTs [40] and in the emulsion polymerization of MMA/BA with graphene filler [41]. The second reason for such a high gel content may be the H-bonding created between the PVP-stabilized MWCNTs and the OH groups of the polymer (due to presence of HEMA) upon film formation.

### Characterization of films cast from hybrid latexes

**Figure 3:**
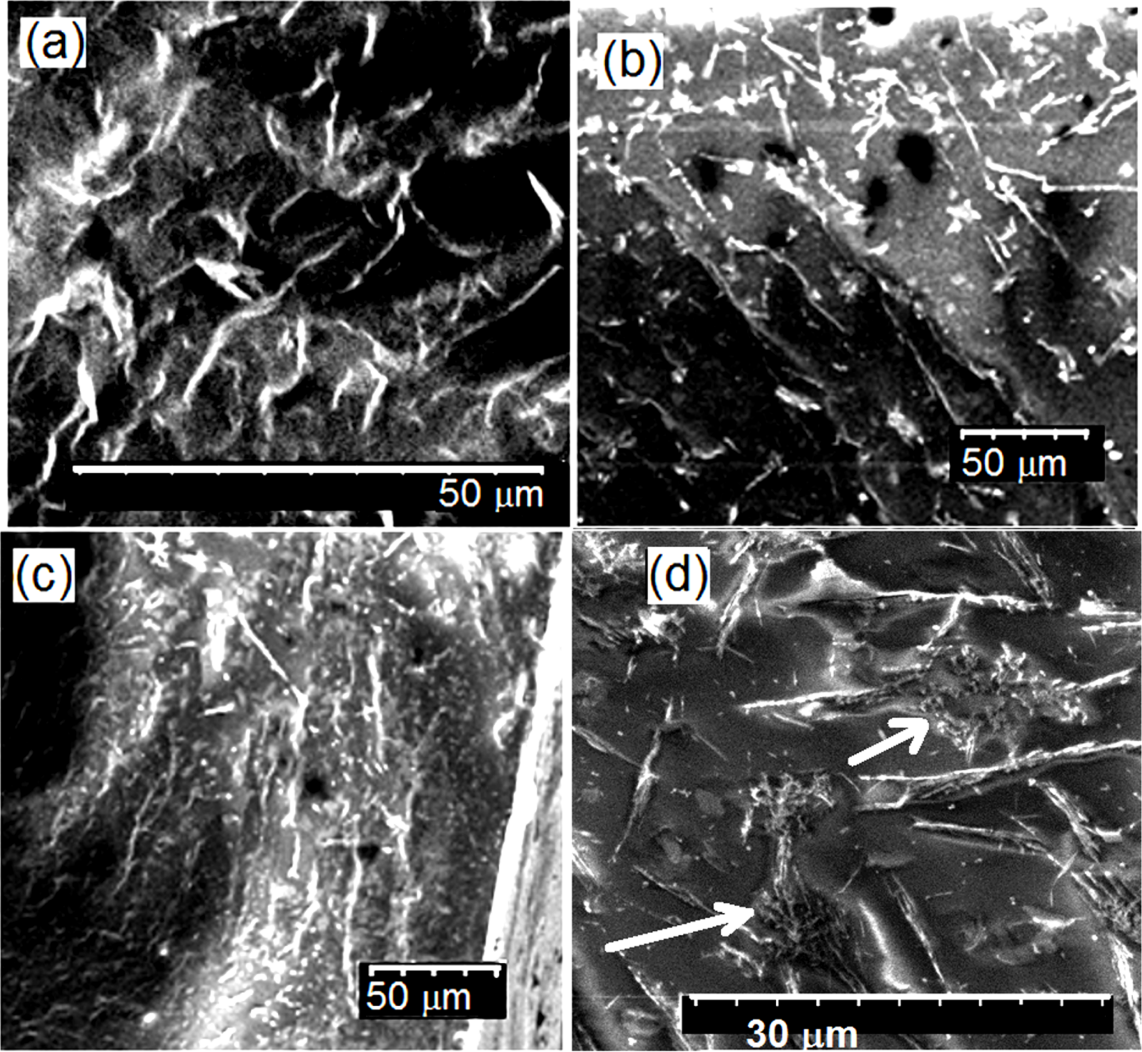
SEM images of the fractured surface of films made of MMA/BA/HEMA/MWCNT in situ hybrid latexes at different air-sonicated MWCNT loadings: (a) 0.1 wt % MWCNT; (b) 0.5 wt % MWCNT; (c,d) 1 wt % MWCNT under different magnifications.

SEM images of the fractured surface of the composite films cast from hybrid latexes at different air-sonicated MWCNT loads are presented in Figure 3, where MWCNTs appear as white structures embedded within a dark polymer matrix. A homogeneous dispersion of the MWCNTs within the matrix is evident at all MWCNT loadings and the presence of larger aggregates may be observed for 1 wt % MWCNTs in Figure 3c,d d (indicated by white arrows in Figure 3d, under higher magnification).

In Table 3, the glass transition temperatures (*T*_g_) of the neat polymer and the polymer composites determined by DSC are presented. The neat polymer and simple blends of the neat polymer and the air-sonicated MWCNTs were used as reference samples. In all the samples, three main transition regions were observed, which are the result of the heterogeneous composition of the polymer formed in the batch polymerization of monomers with different reactivity ratios (*r*_MMA_ = 2.02 ± 0.36, *r*_BA_ = 0.26 ± 0.14) [42] that yield a MMA-rich polymer (*T*_g_ = 90 ºC) at the beginning of the process and an acrylate-rich copolymer at the end. The *T*_g_ of this copolymer was close to −70 ºC, which indicates that it is a copolymer of BA (−54 ºC) and SA (*T*_g_ = −111 ºC).

**Table 3:** Glass transition temperatures of films made of in situ and blends of poly(MMA/BA/HEMA) polymers at different air-sonicated MWCNT concentrations.

MWCNT content	*T*_g1_ region	*T*_g2_ region	*T*_g3_ region

0%	−70 °C	−40 to 50 °C	93 °C
blend 0.50%	−71 °C	−45 to 60 °C	92 °C
blend 1.0%	−71 °C	−45 to 60 °C	90 °C
in situ 0.50%	−71 °C	−45 to 75 °C (additional *T*_g_ at about 50 °C )	90 °C
in situ 1.0%	−69 °C	−45 to 75 °C (additional *T*_g_ at about 50 °C )	90 °C

The broad peak from 40 ºC to about 50 ºC for the neat polymer (Figure S2, Supporting Information File 1) corresponds to the change of the copolymer composition during polymerization. In the case of the blends, this broad peak shifted 10 ºC towards higher temperatures, which denotes that the mobility of the polymer chains was influenced by the presence of the MWCNTs. This, in turn, suggests significant mutual interaction. The PVP used to stabilize the MWCNTs may play a key role in those effects as it is expected to develop π–π interactions with the MWCNTs and hydrogen bonding with the O–H functionalities of the polymer.

In the in situ produced composites, the broad, middle peak shifted to an even higher temperature with the new peak centered at about 50 ºC. This indicates the presence of a new phase that was attributed to grafted polymer, which also contributed to the high fraction of insoluble polymer (see above). The stronger interaction between the polymer and the MWCNTs for in situ composites is further supported by the fact that aging did not vary the results of the DSC measurements, whereas for the neat polymer samples and blends, the 90 ºC peak disappeared through microphase mixing (Figure S2, Supporting Information File 1).

**Figure 4:**
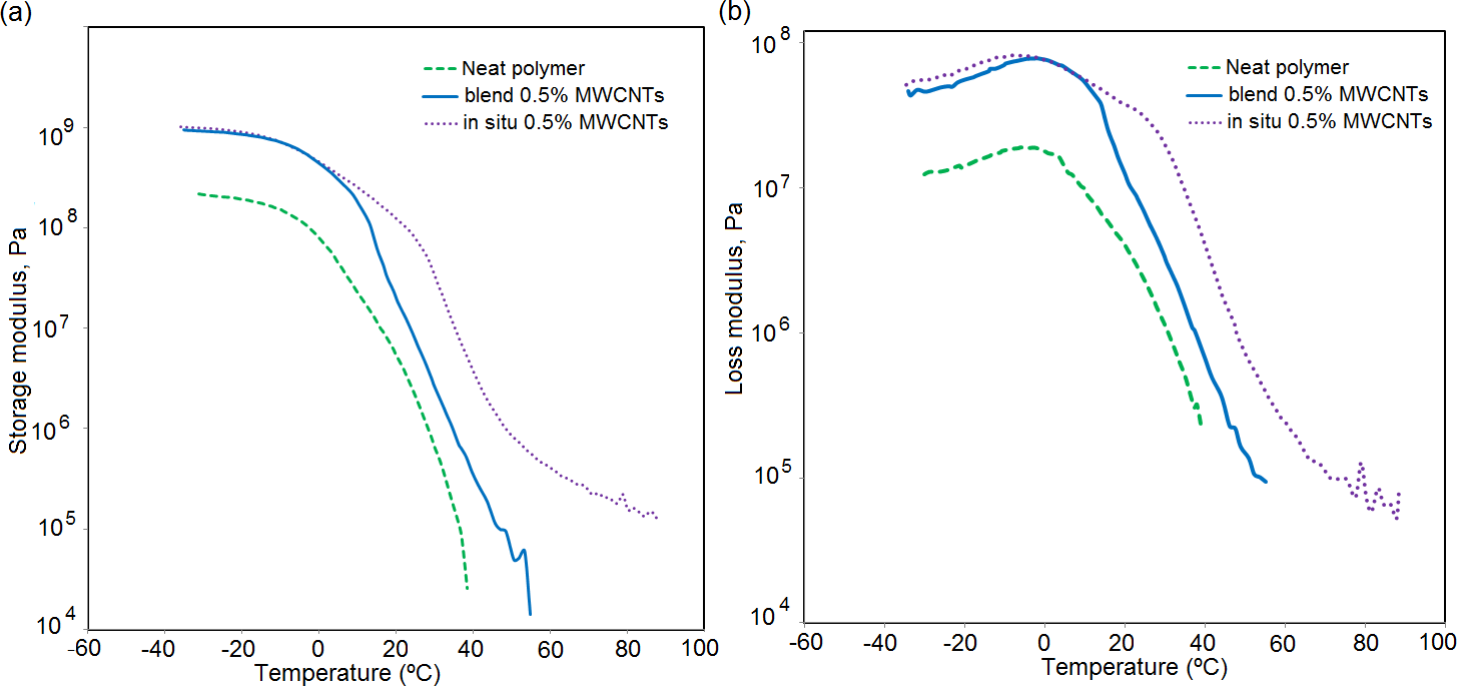
(a) Storage modulus and (b) loss modulus of the films made of MMA/BA/HEMA/air-sonicated MWCNT.

Figure 4a shows that the addition of MWCNTs (0.5 wt %) to the polymer resulted in an augmentation of the storage modulus (i.e., stiffness) over the entire temperature range. In addition, the loss modulus of the composites was also higher than that of the blank polymer (Figure 4b), namely the energy dissipation as heat was promoted. This may be due to an additional energy dissipation mechanism when the MWCNTs slide at the interface with polymer in presence of PVP, as previously reported in case of organic/inorganic hybrids [43].

The strong polymer–MWCNT interaction substantially improves the mechanical properties of the in situ composites, particularly at high temperatures. Figure 4 shows that, whereas in the glassy state (*T* < 20 °C) there was no difference between the blends and in situ composites, in the rubbery region, both the storage and the loss moduli were higher for the in situ composite. The effect was particularly noticeable above 60 ºC. The high moduli of the in situ composites in the high temperature region suggest the formation of 3D networks of the filler within the polymer matrix and significant crosslinking between the both phases [18].

**Figure 5:**
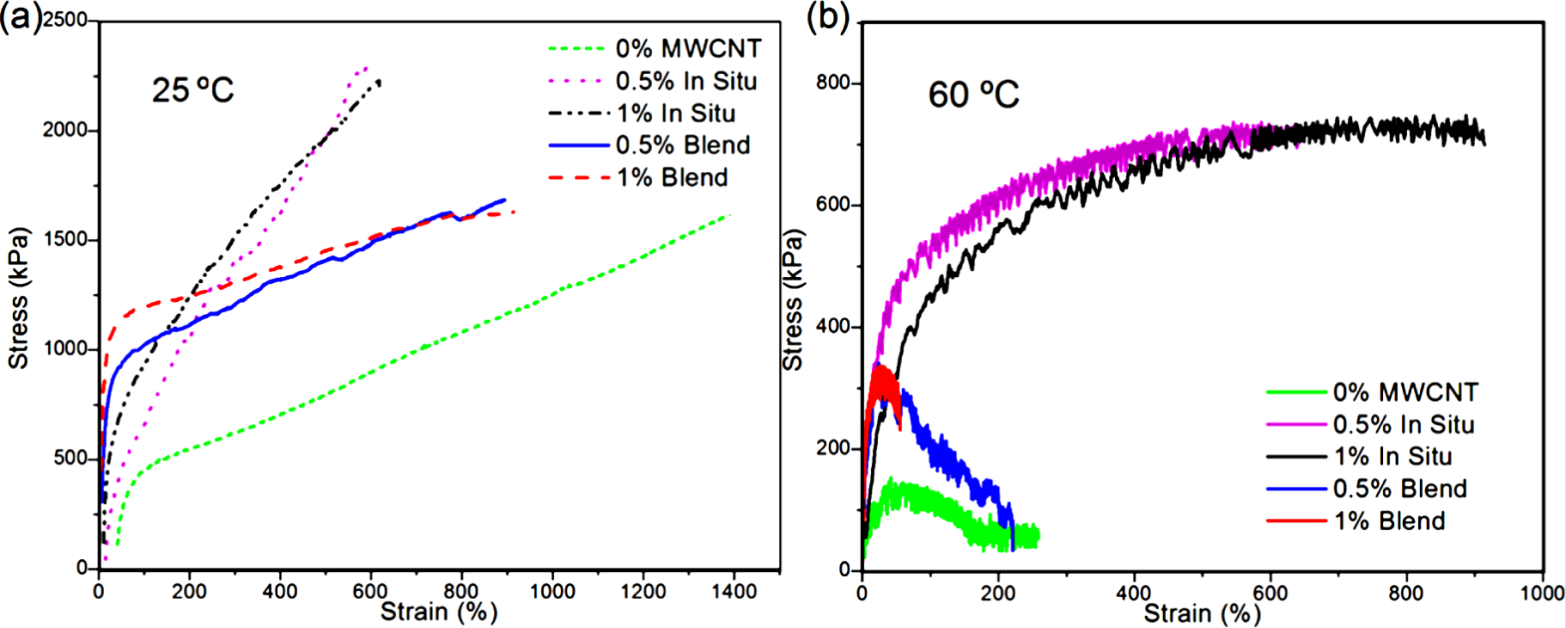
Stress–strain behavior of MWCNT/polymer composites (a) at 25 ºC and (b) at 60 ºC.

In order to gain deeper insight into the reinforcement effect of the MWCNTs in these composites, stress–strain testing of the films was performed at 25 ºC and at 60 ºC (Figure 5).

At 25 ºC, the addition of MWCNTs led to a substantial reinforcement of the polymer with significant differences between blends and in situ composites. Whereas the blends showed a high Young’s modulus followed by a softening after the yield point, the in situ components presented a lower Young’s modulus with a gradual transition from elastic to plastic behavior. In addition, they had a much higher stress at break.

The differences between blends and in situ composites were more acute in the tensile tests carried out at 60 ºC, where the in situ composites maintained the reinforcement, but the neat polymer and the blends behaved as liquid-like materials. As the amount of MWCNTs is the same in the blend and in the in situ composites, the reinforcement was clearly due to the improved interaction between MWCNTs and the polymer, and due to the formation of the 3D reinforcing network of MWCNTs within the matrix.

## Conclusion

Film-forming polymer–MWCNT composite dispersions were synthesized in situ by miniemulsion polymerization of MMA/BA/HEMA/SA in the presence of varying amounts of air-sonicated MWCNTs, stabilized in aqueous dispersions by means of PVP. The reactions proceeded smoothly and resulted in stable, colloidal, hybrid latexes without coagulum. The presence of the MWCNTs had no significant effect on polymerization kinetics, but strongly increased the fraction of insoluble polymer that was attributed to the interaction between the OH groups of the copolymer and the PVP-stabilized MWCNTs and as well to the possible grafting of polymer chains onto MWCNTs. Because monomers with different reactivity ratios were polymerized in batch, a heterogeneous copolymer showing different *T*_g_ values was obtained. The in situ composites showed the presence of an additional phase likely formed as a result of the strong interactions between polymer and MWNCTs (including grafting) that reduced the mobility of the polymer chains.

The MWCNTs were homogeneously dispersed within composite films formed from the hybrid latexes up to a load of 1 wt % MWCNTs, where the presence of larger aggregates was noticed. The performance of the in situ composites was compared with that of the neat polymer dispersion, as well as with those of polymer/MWCNT physical blends. The addition of MWCNTs resulted in a substantial increase of both the storage and the loss moduli. At 60 ºC, which is above the main transition region of the polymer, the in situ composites maintained the reinforcement, whereas the blends behave as a liquid-like material. This suggests the formation of a 3D network in good agreement with the high content of insoluble polymer in the in situ composites.

## Supporting Information

File 1 Colloidal stability of the latexes and the aging effect on the stability of the composite films.
Colloidal stability of polymer and hybrid (in situ 1 wt % MWCNT) latexes, measured by light backscattered from the dispersions, is shown. Differential scanning calorimetry (DSC) results for neat polymer and composite in situ and blend with 0.5 wt % MWCNT are presented for as-received and aged latexes (for three years).
