# Supplementary material for: Miniemulsion copolymerization of (meth)acrylates in the presence of functionalized multiwalled carbon nanotubes for reinforced coating applications
Source: Beilstein J Nanotechnol. 2017 Jun 27;8:1328–37. doi: 10.3762/bjnano.8.134 (PMC5496581; doi:10.3762/bjnano.8.134)
Supplement: File 1 — Colloidal stability of the latexes and the aging effect on the stability of the composite films. Colloidal stability of polymer and hybrid (in situ 1 wt % MWCNT) latexes, measured by light backscattered from the dispersions, is shown. Differential scanning calorimetry (DSC) results for neat polymer and composite in situ and blend with 0.5 wt % MWCNT are presented for as-received and aged latexes (for three years). [file Beilstein_J_Nanotechnol-08-1328-s001.pdf]

Supporting Information

for

Miniemulsion copolymerization of (meth)acrylates in the presence of functionalized multiwalled carbon nanotubes for reinforced coating applications

Bertha T. Pérez-Martínez[1], Lorena Farías-Cepeda[2*], Víctor M. Ovando-Medina[3], José M. Asua[1], Lucero Rosales-Marines[2], and Radmila Tomovska[1,4*]

Address: [1]POLYMAT and Departamento de Química Aplicada, Facultad de Ciencias Químicas, University of the Basque Country UPV/EHU, Joxe Mari Korta zentroa, Tolosa Etorbidea 72, Donostia-San Sebastián 20018, Spain, [2]Departamento de Ingeniería Química, Universidad Autónoma de Coahuila, Blvd. V. Carranza e Ing. José Cárdenas V. S/N, Saltillo, Coah, 25280 México, [3]Ingeniería Química, Coordinación Académica Región Altiplano (COARA) Universidad Autónoma de San Luis Potosí, Carretera a Cedral KM 5+600, San José de las Trojes, Matehuala, SLP, 78700 México, and [4]IKERBASQUE, Basque Foundation for Science, Bilbao, Spain

* Corresponding author

Email: Lorena Farías-Cepeda - lorenafarias@uadec.edu.mx; Radmila Tomovska - radmila.tomovska@ehu.es

Colloidal stability of the latexes and the aging effect on the stability of the composite films

1. Stability of the final latexes with air sonicated MWCNTs

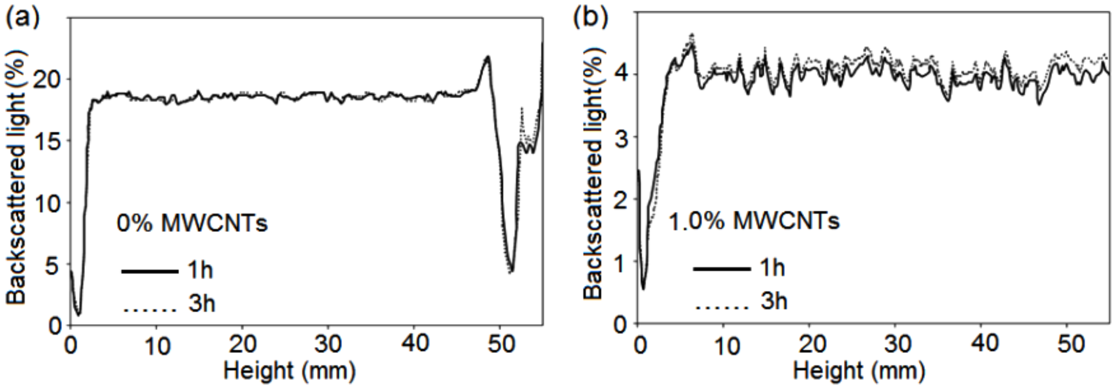

Figure S1. Backscattering analysis at 30 °C of latexes (a) 0% of MWCNT and (b) 1.0% of MWCNT at different times.

Latexes stability was studied by measuring the time evolution of the light backscattered at different heights by the final latexes. Figure S1 presents this evolution for the latex obtained in absence of MWCNTs (Figure S1a) and with 1% of MWCNTs (Figure S1b). Both latexes showed a good stability as no change in the backscattered light signal was noticed in 3 h.

2. DSC results

In Figure S2 the aging results of the polymer and composite films obtained by DSC measurements are presented, as a function of derivative heat flow as on temperature for the neat polymer, the blend (0.5 wt% MCNT) and the in situ composite (0.5 wt% MWCNT).

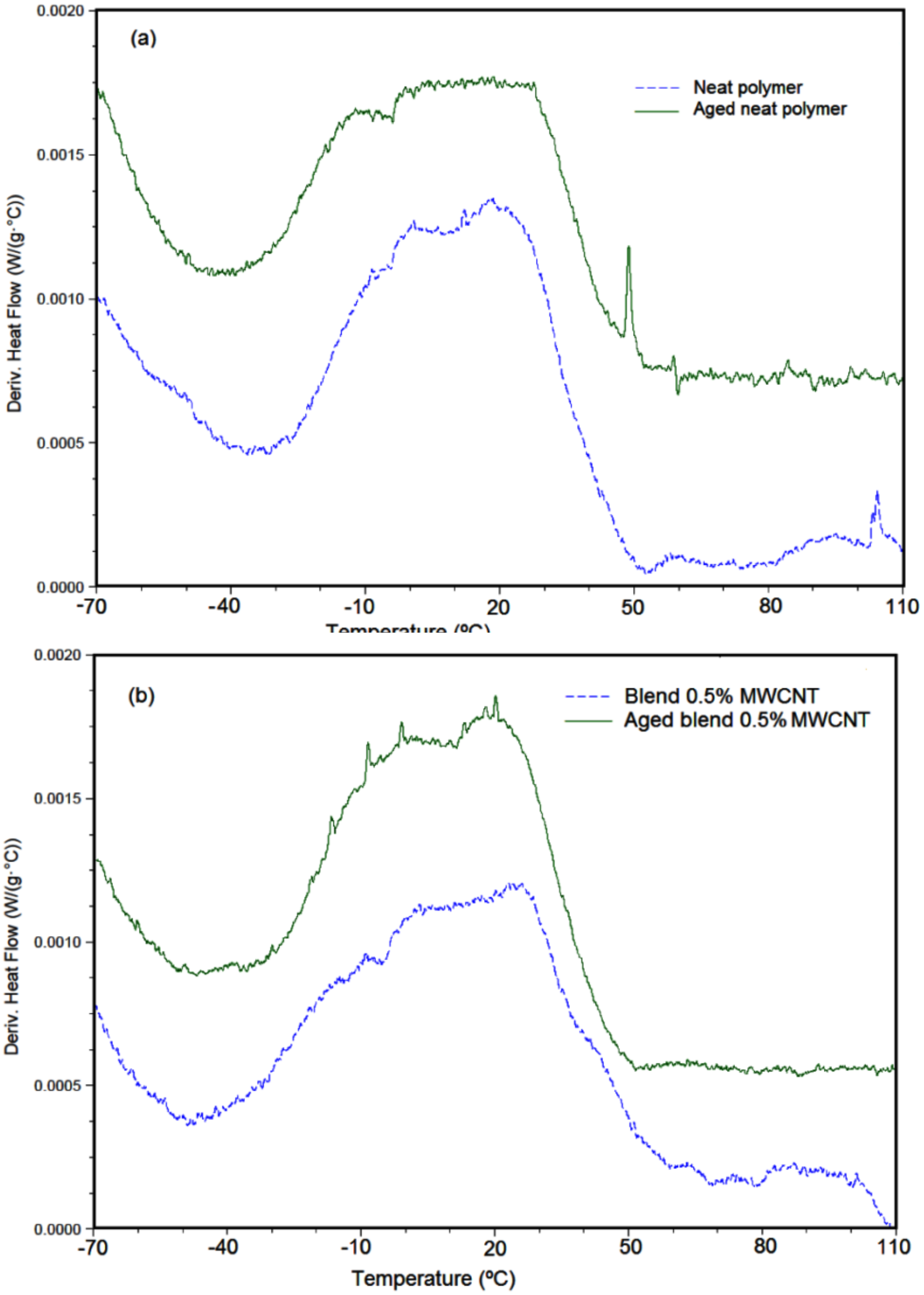
(a)
Neat polymer
Aged neat polymer
Deriv. Heat Flow (W/(g·°C))
0.0020
0.0015
0.0010
0.0005
0.0000
-70
-40
-10
20
50
80
110
(b)
Blend 0.5% MWCNT
Aged blend 0.5% MWCNT
Deriv. Heat Flow (W/(g·°C))
Temperature (ºC)

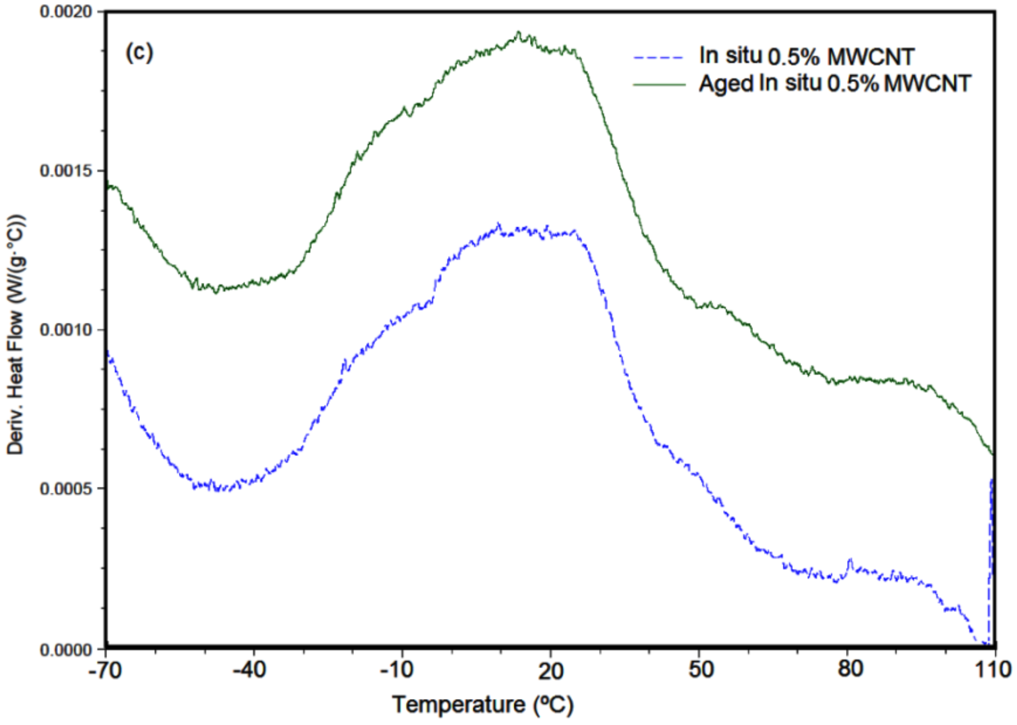

Figure S2. DSC results, Derivative heat flow as a function of temperature for (a) neat and aged neat polymer; (b) blend and aged blend with 0.5 wt% MWCNTs; and (c) in situ and aged in situ with 0.5 wt% MWCNTs.
